# Neuroanatomy and sex differences of the lordosis-inhibiting system in the lateral septum

**DOI:** 10.3389/fnins.2014.00299

**Published:** 2014-09-17

**Authors:** Shinji Tsukahara, Moeko Kanaya, Korehito Yamanouchi

**Affiliations:** ^1^Division of Life Science, Graduate School of Science and Engineering, Saitama UniversitySaitama, Japan; ^2^Department of Human Behavior and Environment Sciences, Faculty of Human Sciences, Waseda UniversitySaitama, Japan

**Keywords:** lordosis, lateral septum, midbrain central gray, estradiol, sexual differentiation

## Abstract

Female sexual behavior in rodents, termed lordosis, is controlled by facilitatory and inhibitory systems in the brain. It has been well demonstrated that a neural pathway from the ventromedial hypothalamic nucleus (VMN) to the midbrain central gray (MCG) is essential for facilitatory regulation of lordosis. The neural pathway from the arcuate nucleus to the VMN, via the medial preoptic nucleus, in female rats mediates transient suppression of lordosis, until female sexual receptivity is induced. In addition to this pathway, other regions are involved in inhibitory regulation of lordosis in female rats. The lordosis-inhibiting systems exist not only in the female brain but also in the male brain. The systems contribute to suppression of heterotypical sexual behavior in male rats, although they have the potential ability to display lordosis. The lateral septum (LS) exerts an inhibitory influence on lordosis in both female and male rats. This review focuses on the neuroanatomy and sex differences of the lordosis-inhibiting system in the LS. The LS functionally and anatomically links to the MCG to exert suppression of lordosis. Neurons of the intermediate part of the LS (LSi) serve as lordosis-inhibiting neurons and project axons to the MCG. The LSi-MCG neural connection is sexually dimorphic, and formation of the male-like LSi-MCG neural connection is affected by aromatized testosterone originating from the testes in the postnatal period. The sexually dimorphic LSi-MCG neural connection may reflect the morphological basis of sex differences in the inhibitory regulation of lordosis in rats.

## Introduction

**Figure 1:**
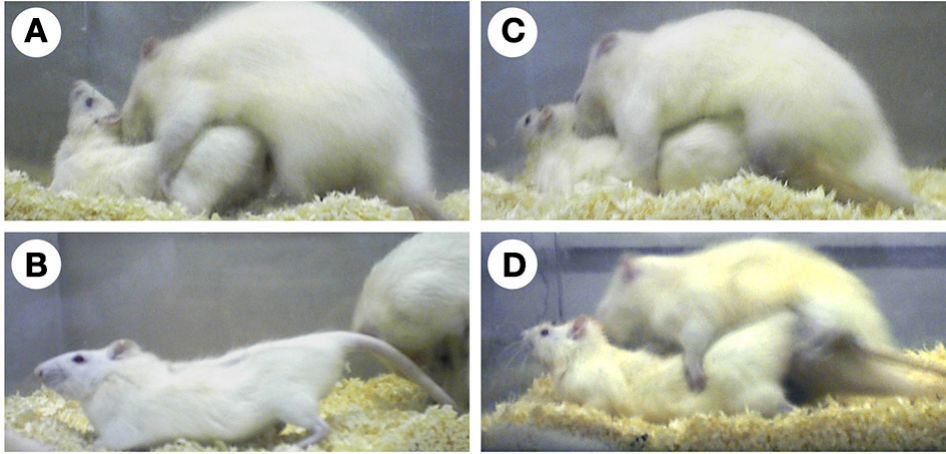
**Photographs of rats displaying sexual behaviors**. An estradiol-treated ovariectomized female rat displays lordosis in response to the mounting of a vigorous male rat **(A,B)**. An estradiol-treated castrated male rat does not exhibit lordosis **(C)**. However, an estradiol-treated castrated male exhibits lordosis when the lateral septum is surgically destructed **(D)**.

Sexual behaviors enable mammals to copulate with the opposite sex and ensure fertilization and consequently reproductive success. One of the most studied sexual behaviors in female mammals is lordosis. Lordosis is a characteristic sexually receptive behavior in female rodents, and this is a postural reflex with dorsiflexion of the vertebral column. The lordosis reflex is observed in sexually receptive female rodents, when their flank perineum region is stimulated by mounting of a vigorous male rodent (Figures 1A,B). Sexual receptive activity of female rodents is modulated by ovarian sex steroids and changes with the estrous cycles: estrous females frequently display lordosis, while anestrous females rarely display lordosis.

As well as anestrous female rats, intact male rats rarely exhibit lordosis. Moreover, most male rats (approximately 88%) do not display lordosis, even when castrated and treated with ovarian sex steroids in adulthood (Yamanouchi and Arai, 1976) (Figure 1C). Although some estradiol benzoate (EB)- and progesterone-treated castrated male rats (approximately 12%) display lordosis, the lordosis quotient (LQ: number of lordosis/number of mounts × 100) is very low (LQ: approximately 10). However, in laboratory rats, lordosis of male rats can be elicited by lesioning of some brain regions and treatment with a large amount of exogenous estradiol. The lateral septum (LS) is one such region, which when lesioned induces lordosis in male rats (Figure 1D). The incidence of lordosis in estradiol-17β (E_2_)- or EB-treated castrated male rats can be increased by surgical destruction of the LS (Nance et al., 1975b; Kondo et al., 1990). Thus, the LS suppresses heterotypical sexual behavior in male rats. Furthermore, this finding supports the idea that the male brain has the potential ability to exhibit sexual behavioral patterns of the opposite sex.

The LS of female rats, as well as male rats, plays an inhibitory role in the regulation of lordosis. Lesioning of the LS enhances lordotic activity induced by EB in ovariectomized female rats (Nance et al., 1975a; Gorzalka and Gray, 1981). Direct implantation of E_2_ into the LS potentiates lordosis in female rats that have been ovariectomized and treated with EB at a subthreshold dose for increasing sexual receptivity; however, the same hormonal manipulation did not induce lordosis in castrated male rats (Satou and Yamanouchi, 1999). This finding indicates that the function of LS in the inhibition of lordosis differs between sexes with respect to responsiveness to estradiol. Thus, inhibitory regulation of lordosis by the LS contributes to estradiol-dependent control of sexual receptivity in female rats and in the suppression of heterotypical sexual behavior in male rats. Understanding the mechanisms responsible for inhibitory regulation of lordosis by the LS will contribute to our understanding of female reproduction and the sexual differentiation of reproductive behaviors in rodent models.

## Estradiol, a key molecule for modulation of female sexual receptivity

Female rats normally exhibit a 4- or 5-day estrous cycle, and female sexual behaviors are displayed during a limited period from the evening of the day of proestrus to the morning of the day of estrus. Estradiol, the levels of which change throughout the estrous cycle, is a key molecule for modulation of female sexual receptivity. Levels of estradiol are high during proestrus because of increasing production of estradiol in the preovulatory ovarian follicle. This leads to an increase in sexually receptive activity in female rodents and the induction of lordosis. Ovariectomy drastically reduces the sexual receptivity of female rats. Decreased receptivity can be subsequently increased by injection of EB or E_2_ (Davidson et al., 1968; Dohanich and Clemens, 1983; Blasberg and Clark, 1997; Tsukahara and Yamanouchi, 2001). One of the important actions of estradiol to induce female sexual receptivity is to activate the facilitatory neural system for lordosis. Neural projections from the ventromedial hypothalamic nucleus (VMN) to the midbrain central gray (MCG) are a critical part of the facilitatory neural system for lordosis (Pfaff et al., 1994, 2008). The VMN expresses estrogen receptors (ERs), transducing estrogen signaling to neural signaling, and then modulating MCG functions to facilitate lordosis (Flanagan-Cato, 2011).

Estradiol evidently acts to increase female sexual receptivity. However, female sexual receptivity is not increased immediately after estradiol affects the brain. In the case of ovariectomized female rats injected with EB followed by progesterone 4 h before testing lordosis, at least 30 h is needed after EB injection to observe the full display of lordosis (Sinchak and Micevych, 2001). Regarding E_2_ or EB treatment alone, more than 6 days are needed to induce sexual receptivity in ovariectomized rats (Dohanich and Clemens, 1983; Blasberg and Clark, 1997; Tsukahara and Yamanouchi, 2001). The delayed effects of estradiol are considered to be due to the genomic actions of estradiol to promote protein synthesis, which is requisite to induce female sexual receptivity. Estradiol-induced expression of progesterone receptors requires approximately 16 h following estradiol treatment, and this estradiol-induced expression is required for progesterone to exert its facilitatory effects on lordosis (Parsons et al., 1979, 1980, 1981). Another explanation for the delayed effects of estradiol on female sexual receptivity is that estradiol initially suppresses lordotic activity until female rats show a maximal level of female sexual receptivity. Recently, Micevych and his colleagues proposed a neural system that is activated by rapid actions of estradiol via membrane signaling, resulting in transient suppression of lordosis in female rats (Micevych and Christensen, 2012; Micevych and Sinchak, 2013). Reportedly, estradiol acts rapidly through estradiol membrane signaling to release neuropeptide Y in the arcuate nucleus of the hypothalamus. Subsequently, there is activation of β-endorphin neurons, which express neuropeptide Y-Y1 receptors, which project from the arcuate nucleus to the medial preoptic nucleus. In the medial preoptic nucleus, neurons expressing μ-opioid receptors and projecting to the VMN are stimulated by β-endorphin, resulting in the inhibition of lordosis. Thus, transitory inhibition of lordotic activity by rapid actions of estradiol may be necessary for estrous female rats to exhibit full performance of lordosis.

On diestrous and estrous days, when the levels of estradiol in the blood are low, female rats rarely display lordosis, even if male rats attempt copulation. One reasonable explanation for decreased sexual receptivity during the anestrous phase is that the facilitatory neural system for lordosis is not activated in the absence of certain estradiol levels. In addition, the inhibitory neural system may contribute to the control of sexual receptivity in female rats. Although the detailed mechanisms responsible for inhibition of lordosis are poorly understood, several regions involved in the inhibitory regulation of lordosis have been documented, including the LS, medial preoptic nucleus, and dorsal raphe nucleus (Yamanouchi, 1997). Here, we focus on the lordosis-inhibiting system in the LS, as discussed below.

## Lordosis-facilitating system: a common pathway from the VMN to the MCG

The VMN is known as an important component of the facilitatory neural system for lordosis. Surgical destruction of the VMN in female rats prevents the display of lordosis (Mathews and Edwards, 1977; Pfaff and Sakuma, 1979a), while electrical stimulation of the VMN facilitates lordosis (Pfaff and Sakuma, 1979b). Injection of EB into the VMN stimulates ovariectomized female rats to display lordosis (Barfield and Chen, 1977). The VMN of the rat brain, especially the ventrolateral part of the VMN (VL-VMN), abundantly expresses estrogen receptor-α (ERα) but not ERβ (Shughrue et al., 1997; Osterlund et al., 1998). The actions of estrogens binding to ERα are essential for the induction of lordosis behavior. This is illustrated by studies showing that lordosis is elicited in ovariectomized female rats by injection of a selective ERα agonist, but not a selective ERβ agonist (Mazzucco et al., 2008). Female mice lacking the ERα gene do not show any lordosis response (Ogawa et al., 1998). In contrast, sexual behaviors of ERβ-knockout female mice are indistinguishable from those of wild-type female mice (Ogawa et al., 1999). Sexually receptive behaviors in female mice are abolished by knockdown of ERα in the VL-VMN (Musatov et al., 2006), suggesting that ERα expressed in this region is necessary to induce lordosis behavior. Thus, the VMN exerts an estrogen-dependent facilitatory influence on the control of lordosis in female rodents.

Some VMN neurons project axons to the MCG, and neural projections from the VMN to MCG are an important part of the neural circuitry underlying lordosis (Daniels et al., 1999). The lordosis response in female rats disappears after lesion of the MCG (Sakuma and Pfaff, 1979b), while it is activated by electrical stimulation of the MCG (Sakuma and Pfaff, 1979a). Electrical stimulation of the VMN facilitates lordosis in female rats, but this effect is nullified by MCG lesions (Sakuma and Pfaff, 1979b). Transection of neural connections between the VMN and MCG results in elimination of the lordosis response in female rats (Hennessey et al., 1990). Neurons of the MCG project axons to the medullary reticular formation of the hindbrain, which controls motoneurons in the lumbar spinal cord, thus innervating axial muscles involved in maintaining the lordosis posture (Pfaff et al., 1994). According to a study demonstrating the central neural circuit innervating the lumber epaxial muscle in female rats, neurons comprising the circuit were concentrated in the ventrolateral column, rather than the dorsal, dorsolateral, or ventral columns of the MCG, and were mainly localized in the VL-VMN rather than other parts of the VMN (Daniels et al., 1999). Thus, neural projections from the VL-VMN to the ventrolateral column of the MCG form a critical neural pathway for facilitatory regulation of lordosis. Interestingly, most VMN neurons projecting to the MCG do not express ERα (Calizo and Flanagan-Cato, 2003). However, the VMN contains many ERα-expressing cells (Shughrue et al., 1997; Osterlund et al., 1998) and ERα expressed in the VMN plays a facilitatory role in lordosis induction (Musatov et al., 2006). After mating, of those VMN neurons expressing Fos, a marker for neuronal activation, approximately 41% neither contain ERα nor project to the MCG, and 35% contain ERα but do not project to the MCG (Calizo and Flanagan-Cato, 2003). Flanagan-Cato proposed that there are at least three different types of VL-VMN neurons participating in the control of lordosis behavior: ERα-containing neurons that may serve as local circuit neurons, MCG-projecting neurons without ERα, and neurons that neither express ERα nor project to the MCG, but are activated during mating (Flanagan-Cato, 2011). Local neural circuitry comprising these neurons in the VL-VMN could underlie lordosis-facilitating functions of the VMN, which detects estrogens and modulates the functions of the MCG. Besides neural projection from the VL-VMN, the MCG also receives neural information from other regions, including the habenular nucleus, medial amygdala, and zona incerta (Beitz, 1995). These brain regions are reported to have facilitatory influences on the regulation of lordosis in female rats (Modianos et al., 1975; Dornan et al., 1991; Rajendren and Moss, 1993). Additionally, the MCG receives sensory information from the flank skin stimulated by male mounting via the lumber spinal cord (Pfaff et al., 1994). Thus, the MCG plays an important role in the integration of neural information from the forebrain, lower brain stem, and spinal cord to regulate lordosis.

## Lordosis-inhibiting system in the LS

### Inhibitory regulation of lordosis by the LS

The LS of the forebrain is known to participate in the control of instinctive behaviors related to fear, aggression, and reproduction. LS lesions cause hyperirritability, hyper reactivity, and hyper aggressiveness in rats (Albert and Wong, 1978; Albert, 1980). In male rats, the LS is involved in the facilitation of male sexual behavior and the inhibition of heterotypical sexual behavior. Lesion of the LS effectively suppresses male sexual behavior (Kondo et al., 1990) and facilitates lordosis behavior in male rats (Nance et al., 1975b; Kondo et al., 1990). Injection of a neurotoxin into the LS also induces lordosis in E_2_-treated castrated male rats (Tsukahara and Yamanouchi, 2001), indicating that neurons localized in the LS themselves function to inhibit lordosis in male rats. The LS exerts an inhibitory influence on lordosis not only in male rats but also in female rats, because lordosis response is enhanced by lesion of the LS in female rats (Nance et al., 1975a; Gorzalka and Gray, 1981) and in female hamsters (Nance and Myatt, 1987), while electrical stimulation in the LS suppresses lordosis behavior in female hamsters (Zasorin, 1975).

**Figure 2:**
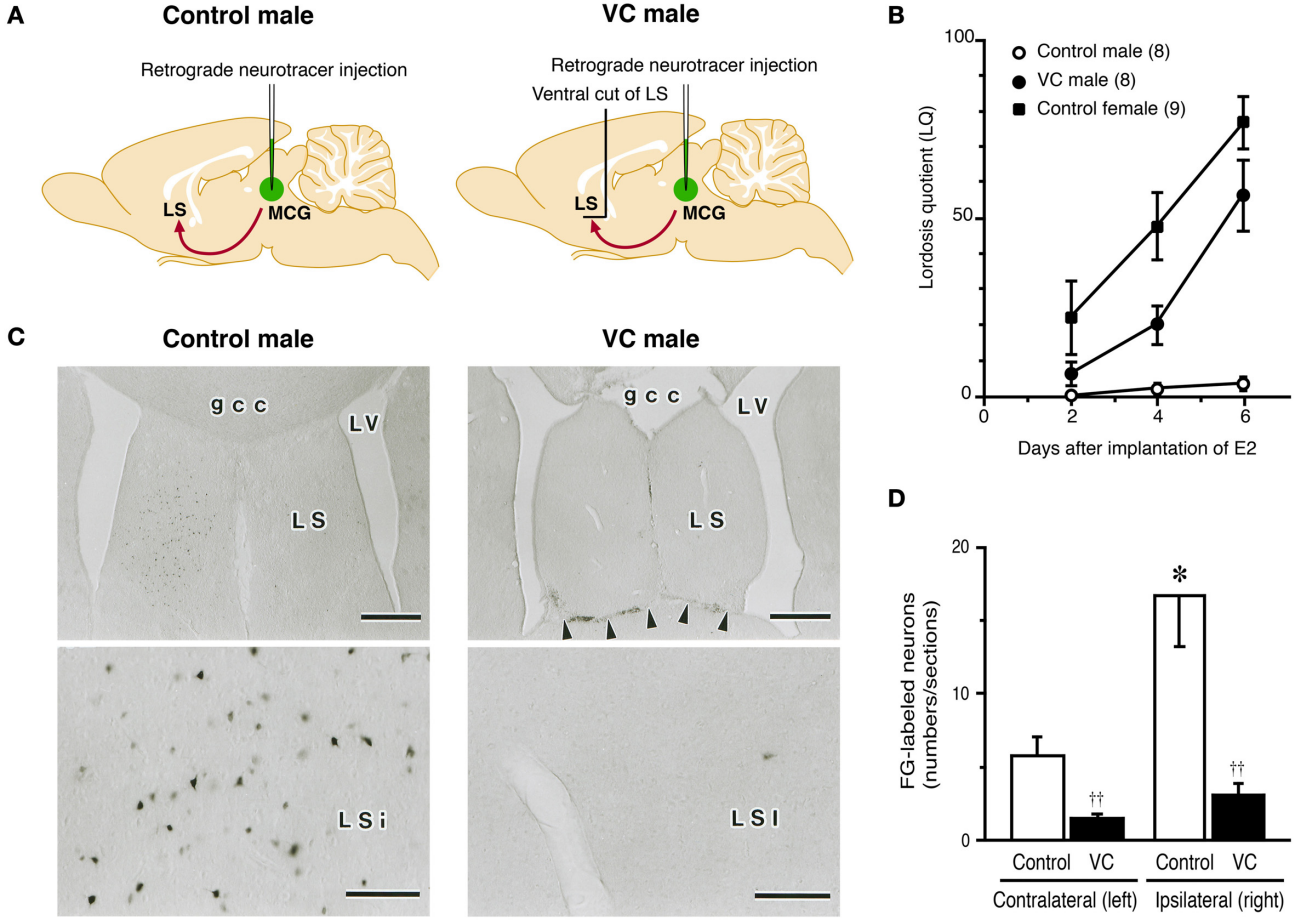
**Effects of a ventral cut of the LS (VC) on lordotic activity and neural projection from the LS to MCG in male rats. (A)** Estradiol-17β (E_2_)-treated castrated male rats with or without VC were tested for lordosis behavior and then injected with Fluoro-Gold (FG), a retrograde neurotracer, into the MCG. FG-labeled neurons in the LS were detected by immunohistochemistry. **(B)** The mean LQ of E_2_-treated castrated male rats with VC (VC male) was increased over time (days) after E_2_ treatment, like an E_2_-treated ovariectomized female (control female), but the mean LQ of E_2_-treated castrated male rats without VC (control male) was low. **(C)** Photomicrographs of the LS of control and VC male rats that received FG injection into the MCG on the right side. Many FG-labeled neurons were found in the LSi of control male rats, but not in the LSi of VC male rats. Arrowheads indicate the scar of VC. Scale bars = 500 μm. gcc, genu of the corpus callosum; LS, lateral septum; LSi, intermediate part of the LS; LV, lateral ventricle; MCG, midbrain central gray. **(D)** The number of FG-labeled neurons in the LSi of control male rats was significantly greater than that of VC male rats on the ipsilateral and contralateral side of the FG injection site. The LSi of control male rats contained many more FG-labeled neurons on the ipsilateral side of the FG injection site. ^††^*p* < 0.01 vs. control male rat; ^*^p < 0.05 vs. contralateral side (modified from Tsukahara and Yamanouchi, 2001).

Although the male LS contributes to suppression of heterotypical sexual behavior, LS lesions alone do not result in full display of lordosis in male rats, as seen in female rats. The level of vertebral column dorsiflexion and the LQ in LS-lesioned male rats are lower than those in sexually receptive female rats (Figures 1A,D, 2B). Accordingly, inhibitory functions of heterotypical sexual behavior are inherent not only in the LS, but also in other brain regions. The medial preoptic nucleus and the dorsal raphe nucleus have an inhibitory influence on lordosis display in male rats (Van De Poll and Van Dis, 1979; Hennessey et al., 1986; Kakeyama and Yamanouchi, 1992). Lesioning of both the LS and dorsal raphe nucleus induces lordosis display in male rats at a comparable level to that of female rats (Kakeyama and Yamanouchi, 1994). Thus, development of the inhibitory neural systems for lordosis in other regions except the LS is also critical for sexual differentiation of sexual behavioral patterns.

It appears that LS neurons involved in the inhibition of lordosis elongate their axons ventrally, because horizontal deafferentation at the site ventral to the LS elicits the lordosis reflex in rats of both sexes (Yamanouchi and Arai, 1977, 1985, 1990). Neural fibers projecting from the LS join the medial forebrain bundle (MFB) (Veening et al., 1982). Transection of the MFB at the suprachiasmatic level enhances lordosis in female rats (Yamanouchi and Arai, 1989) and induces lordosis in male rats (Yamashita Suzuki and Yamanouchi, 1998). Therefore, it is considered that lordosis-inhibiting neurons of the LS terminate at the brain stem after the neural fibers pass through the MFB. The VMN and MCG are possible projection sites for lordosis-inhibiting neurons of the LS, because both are major components of the facilitatory neural system for lordosis, as mentioned above. However, an anatomical study demonstrated that the VMN does not receive direct input from any part of the LS in rats (Risold and Swanson, 1997b). Furthermore, it indicated that the LS and VMN are functionally independent of each other for regulation of lordosis in female rats (Yamanouchi, 1980; King and Nance, 1985). In this context, lordosis-inhibiting neurons of the LS presumably send their axons to other regions than the VMN. There is anatomical evidence for direct neural connections from the LS to MCG in guinea pigs (Staiger and Nurnberger, 1991) and rats (Risold and Swanson, 1997b). The lordosis response in female rats is enhanced by a LS lesion, but this effect disappears when the MCG is surgically destroyed in combination with the LS lesion (Kondo et al., 1993). This finding indicates the possibility that the LS functionally links to the MCG to exert an inhibitory influence on the regulation of lordosis.

### LS-MCG connections for inhibition of lordosis

To clarify whether neural connections between the LS and MCG have a functional role in the inhibition of lordosis, we carried out a neuroanatomical and behavioral study (Tsukahara and Yamanouchi, 2001). We performed behavioral tests for lordosis in E_2_-treated castrated male rats, some of which bore neural transections of the ventral outputs from the LS (Figure 2A). Mean LQ in castrated male rats that received neural transections of the ventral outputs from the LS gradually increased days after implantation of E_2_ (Figure 2B), as reported previously (Yamanouchi and Arai, 1975, 1978, 1985). In contrast, most of the E_2_-treated castrated male rats without neural transection did not display lordosis, and the mean LQ was low throughout behavioral testing. After behavioral testing, we injected Fluoro-Gold (FG), a retrograde neurotracer, into the MCG to determine the location of FG-labeled neurons in the LS and the effects of the neural transections on retrograde transport of FG from the MCG to LS (Figure 2A). In E_2_-treated castrated male rats that exhibited lower performance of lordosis, the intermediate part of the LS (LSi), especially the rostral part of the LSi, contained a large number of FG-labeled neurons (Figures 2C,D). However, other parts of the LS contained only a few FG-labeled neurons. In E_2_-treated castrated male rats that exhibited higher performance of lordosis following neural transections of the ventral outputs from the LS, only a few FG-labeled neurons were found in the LSi. These findings suggest that male rats can display lordosis when the neural projection from the LSi to the MCG is transected.

The LS is classically divided into three parts, the aforementioned LSi, the dorsal LS (LSd), and the ventral LS (LSv) based on the size and density of neurons (Jakab and Leranth, 1995). Of the three parts, the LSi is the largest subdivision and exhibits the most heterogeneous cytoarchitecture, with loosely grouped neurons of different cell sizes. Neurons of the LSi themselves function to suppress the display of lordosis, because E_2_-treated castrated male rats can exhibit lordosis when LSi neurons are completely killed by a neurotoxin, but not when chemical lesion of the LSi is incomplete (Tsukahara and Yamanouchi, 2001). The LS contains several types of neurons that produce neuropeptides, opioid peptides, and gamma-aminobutyric acid (GABA) as neurotransmitters (Risold and Swanson, 1997a; Tsukahara and Yamanouchi, 2003). In the rostral part of the LSi, from which many neurons project to the MCG (Risold and Swanson, 1997b; Tsukahara and Yamanouchi, 2001), neurons produce GABA, neurotensin, or enkephalin (Risold and Swanson, 1997a; Tsukahara and Yamanouchi, 2003). Therefore, these substances are candidate neurotransmitters that may transfer neural information for lordosis-inhibiting neurons of the LSi, but this is yet to be determined. The MCG of rats contains GABA_A_ receptors and GABA_B_ receptors (Williams and Beitz, 1990b; Barbaresi, 2007). Systemic administration of a GABA_A_ receptor agonist or a GABA_B_ receptor agonist inhibits lordosis in rats (Agmo et al., 1989; Luine et al., 1991; Kakeyama and Yamanouchi, 1996). However, local GABAergic neurotransmission via GABA_A_ receptors in the MCG is involved in facilitatory regulation of lordosis (McCarthy et al., 1991; Mccarthy et al., 1994; McCarthy et al., 1995). GABAergic neurons are generally divided into local circuit neurons with short axons and projection neurons with longer axons, and most GABAergic neurons function as local circuit neurons (Vincent et al., 1982; Ottersen and Storm-Mathisen, 1984; Ottersen et al., 1995). GABAergic neurons in the MCG may act as local circuit neurons to mediate facilitatory effects on lordosis, although the roles of GABAergic neurons in the LSi in the regulation of lordosis are largely unknown. Enkephalin may serve as a neurotransmitter in the MCG to inhibit lordosis in female rats and the MCG of rats contains enkephalinergic nerve terminals (Williams and Beitz, 1990a; Beitz, 1995). Lordotic activity of EB- and progesterone-treated ovariectomized female rats is decreased by injection of Met-enkephalin into the MCG in combination with an inhibitor of enkephalin degrading enzymes (Bednar et al., 1987). Neurotensinergic nerve terminals and their receptors are found in the MCG of rats (Shipley et al., 1987; Boudin et al., 1996). However, there is no evidence for the involvement of neurotensin in the regulation of lordosis.

**Figure 3:**
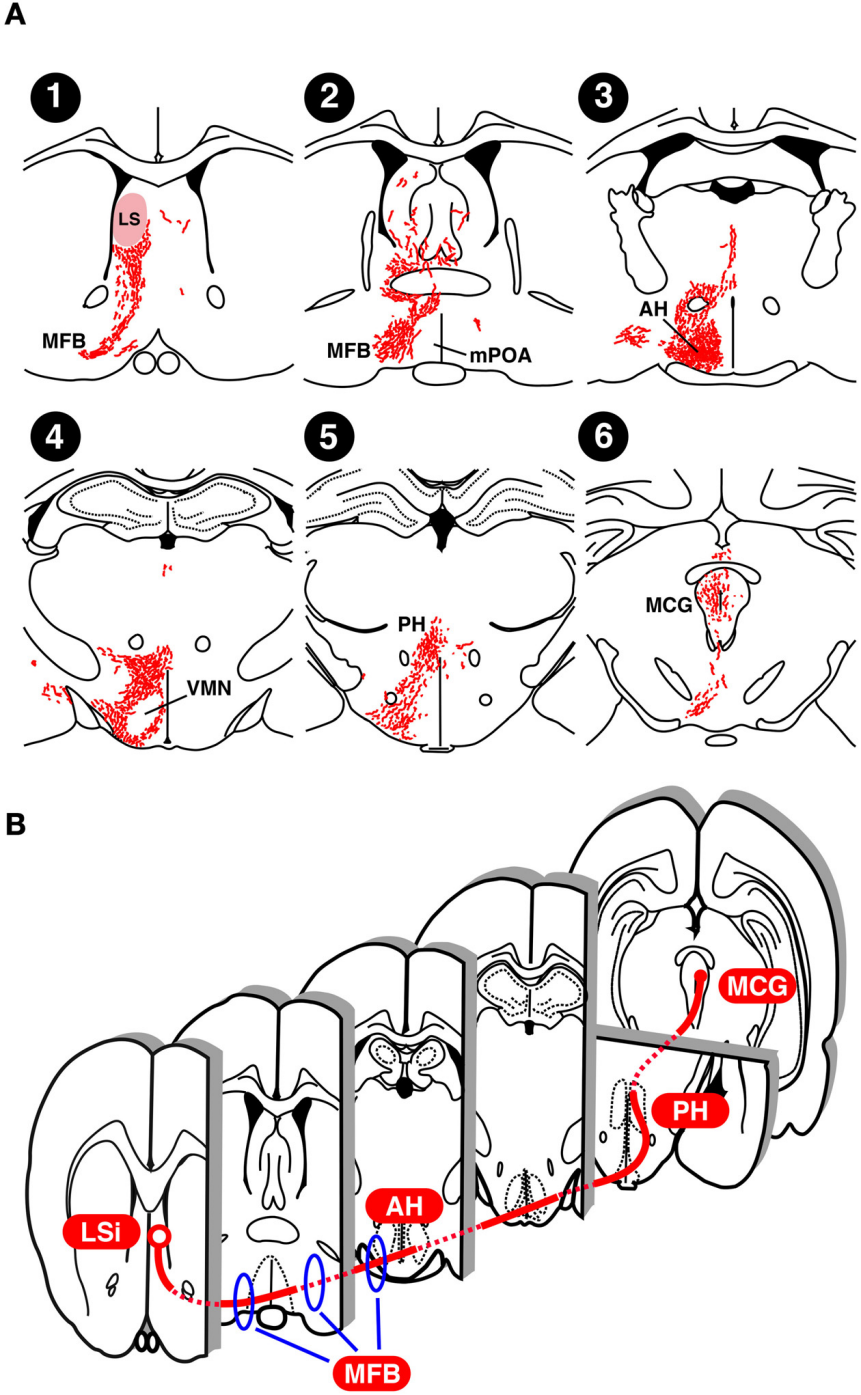
**Neural projection of the LS in rats. (A)** Distribution of Phasiolus vulgaris leucoagglutinin (PHAL), an anterograde neurotracer, -labeled neural fibers in an estradiol-treated castrated male rat that received PHAL injection into the LS on the right side (modified from Tsukahara and Yamanouchi, 2001). **(B)** Possible lordosis-inhibiting neural tract from the LSi to MCG. AH, anterior hypothalamic area; LS, lateral septum; MCG, midbrain central gray; MFB, medial forebrain bundle; mPOA, medial preoptic area; PH, posterior hypothalamic area; VMN, ventromedial hypothalamic nucleus.

**Figure 4:**
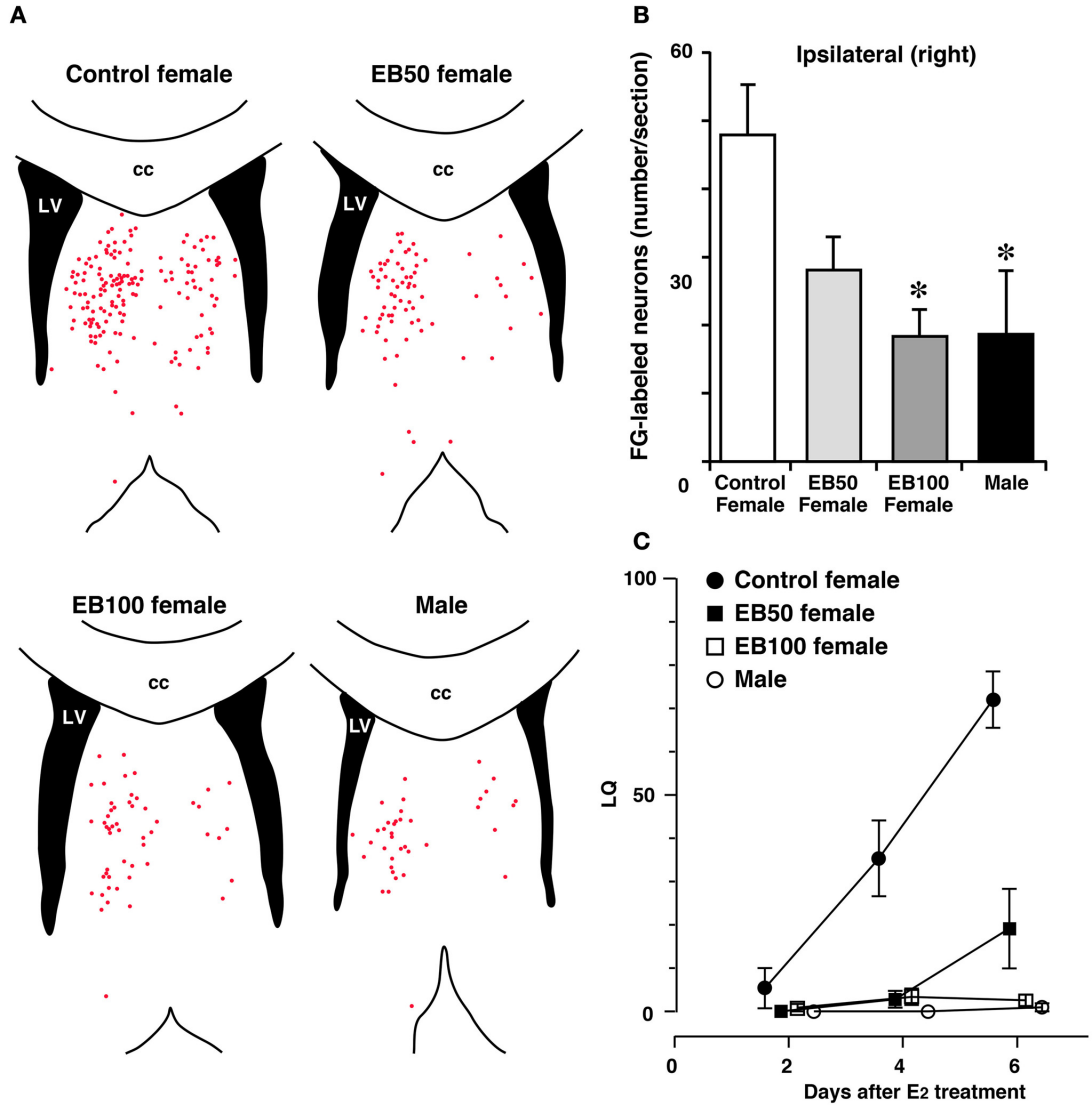
**Sex difference in neural projection from the LSi to MCG and effects of postnatal treatment with estradiol benzoate (EB) in the formation of the neural projection**. Distribution of Fluoro-Gold (FG)-labeled neurons in the LS **(A)** and the number of FG-labeled neurons in the LSi **(B)** of female and male rats that received FG injection into the MCG. Female rats were subcutaneously injected with 50 or 100 μg of EB or vehicle on postnatal day 5 (day 1 = date of birth), and they were ovariectomized and treated with estradiol in adulthood (EB50, EB100, and control female groups). Male rats were castrated and treated with estradiol in adulthood. Each red dot of the line drawings of **(A)** indicates a FG-labeled neuronal cell body. Postnatal EB treatment dose-dependently decreased the number of FG-labeled neurons in the LSi of female rats, resulting in elimination of sex differences in the number of FG-labeled neurons. cc, corpus callosum; LV, lateral ventricle. ^*^p < 0.05 vs. control female. **(C)** The mean LQ of control, EB50, EB100 female rats, and male rats. Postnatal EB treatment dose-dependently decreased lordotic activity of female rats (modified from Tsukahara et al., 2003).

Neurons of the LS send axons to a variety of regions in the thalamus, hypothalamus, and midbrain in a subdivision-specific manner, and the septal region that sends the largest number of axons to the MCG is the rostral LSi (Risold and Swanson, 1997b). To determine the lordosis-inhibiting neural tracts from the LS to MCG, we traced the neural projections from the LSi to the MCG in E_2_-treated castrated male rats using Phaseolus vulgaris leucoagglutinin (PHAL), an anterograde neurotracer (Tsukahara and Yamanouchi, 2001) (Figure 3A). Neural tracts from the LS to MFB (Figures 3A-1,2) are essential for lordosis-inhibiting LSi neurons to function, because the lordosis reflex is induced in male rats by transection of the ventral area of the septal region (Yamanouchi and Arai, 1985) and by transection of the MFB (Yamashita Suzuki and Yamanouchi, 1998). Similar surgical manipulations enhance lordotic activity in female rats (Yamanouchi and Arai, 1977, 1989, 1990). Thus, the MFB includes fibers originating from lordosis-inhibiting LSi neurons in both sexes. After passing through the MFB, PHAL-labeled axonal fibers reach the anterior hypothalamic area (Figure 3A-3). The VMN did not contain any PHAL-labeled axons, but many PHAL-labeled fibers existed in the region surrounding it (Figure 3A-4), supporting previous studies showing that the LS and VMN are functionally independent of each other in the regulation of lordosis (Yamanouchi, 1980; King and Nance, 1985). PHAL-labeled axonal fibers in the posterior hypothalamic area projected along the longitudinal axis from the ventral region (Figure 3A-5), and then terminated at the rostral part of the MCG (Figure 3A-6). It is likely that lordosis-inhibiting LSi neurons elongate their axons to the MCG by passing through the posterior hypothalamic area, because the expression of lordosis in female rats was increased by transection of neural fibers passing through the medial regions, including the posterior hypothalamic area (Ohnishi et al., 2003). Taking these results together, we propose that lordosis-inhibiting neural tracts from the LSi to MCG include the MFB at the level of the optic chiasma, the ventrolateral hypothalamic regions (including the anterior hypothalamic area, and excluding the VMN), and the medial part of the junction of the diencephalon and mesencephalon (including the posterior hypothalamic area) (Figure 3B).

### Sexual differentiation of the lordosis-inhibiting system in the LS

#### Morphological sex difference in the LSi

Nuclei exhibiting morphological differences by sex are generally termed sexually dimorphic nuclei (SDNs) and are found in the central nervous system (Woodson and Gorski, 1999). The LSi is one of the SDNs in the rat brain. The volume of the LSi in prepubertal female rats is larger than that of same-aged male rats, however, there are no sex differences in the volume of the LSd and LSv (Tsukahara et al., 2004). The number of LSi neurons in adult female rats is greater than that in adult male rats, while there are no sex differences in the number of neurons in the LSd and LSv (Segovia et al., 2009). Thus, sexual dimorphism of the LSi is reflected by its larger size and greater number of neurons in female rats than in male rats.

In rodent models, it has been long considered that organizational effect of aromatizable testosterone originating from the testes during the perinatal period is critical for the formation of morphological sex differences in the brain (McEwen et al., 1977; MacLusky et al., 1979; MacLusky and Naftolin, 1981). This is based on the classic concept for understanding the sexual differentiation of the brain by sex steroids, but this view has now been revised. However, testicular testosterone that acts on the brain during the perinatal period is still necessary, but not solely, for the formation of morphological sex differences in the brain. Testosterone synthesis in the testis of rats begins on embryonic day 15.5, rises to a peak around embryonic day 18.5, and then declines after birth (Warren et al., 1973). Temporal change in plasma testosterone levels is similar to that in testosterone synthesis in the testes, and male rats have higher plasma testosterone levels from embryonic day 18 to postnatal day 5 (PD5, day of birth = PD1) than do female rats (Weisz and Ward, 1980). This period corresponds to the classically identified critical period, when testosterone effectively masculinizes and defeminizes the brain (MacLusky and Naftolin, 1981). Testosterone in the postnatal period has masculinizing effects on the morphology of the LSi at least in part, because the number of LSi neurons in male rats is increased by castration on the day of birth (Segovia et al., 2009).

In the currently revised view of brain sexual differentiation, the period when the sexually differentiated brain is organized under the influence of sex steroids is not limited to the perinatal period, but is extended to the pubertal/adolescent period (Schulz et al., 2009a; Juraska et al., 2013). Ahmed et al. reported that new cells generated during puberty are added to the anteroventral periventricular nucleus (AVPV), a female-biased SDN, and the sexually dimorphic nucleus of the preoptic area (SDN-POA) and medial amygdala (Me), male-biased SDNs, and that the cell number and volume of the AVPV in female rats and those of the SDN-POA and Me in male rats are greater than those in the opposite sexes (Ahmed et al., 2008), indicating a significant contribution of cell generation during puberty in the formation of morphological sex differences in the brain. They further showed that gonadectomy at PD20 suppresses the increase in the number of new cells and the volume of the female AVPV and of the male SDN-POA and Me, excepting the volume of the male Me, whereas cell number and volume of the opposite sexes do not change with gonadectomy (Ahmed et al., 2008). Sexually dimorphic formation of the principal nucleus of the bed nucleus of the stria terminalis (BNSTp), another male-biased SDN in rodents, is also affected by gonadal hormones during the prepubertal and/or pubertal period. The number of neurons in the male BNSTp is greater than that in the female BNSTp in 20-day-old prepubertal mice, and this sex difference becomes marked in adulthood with increasing neuron number in the male BNSTp and loss of neurons in the female BNSTp (Wittmann and Mclennan, 2013). These findings indicate that ovarian and testicular hormones during puberty act in remodeling the brain after it develops with or without the organizational effect of testicular testosterone during the perinatal period.

In addition to the organizational effects of sex steroids, sex chromosome genes directly influence the sexual differentiation of the brain (McCarthy and Arnold, 2011; Arnold, 2014; Cox et al., 2014). The expression of tyrosine hydroxylase (TH) in dopamine neurons of the murine midbrain differs by sex, and it is higher in male mice. The higher TH expression is due to *Sry*, which is a dominant testis-determining gene of the mammalian Y chromosome (Lovell-Badge and Robertson, 1990), because the TH expression is reduced by suppression of *Sry* expression (Dewing et al., 2006). Moreover, other sex chromosome genes also contribute to sex differences in TH expression, which is revealed by the four core genotypes model (Carruth et al., 2002). The four core genotypes model consists of mice in which the complement of sex chromosomes (XX vs. XY) is made independently of gonadal sex (Arnold and Chen, 2009). It was revealed by studies using the four core genotypes model that vasopressin neural fibers in the LS, which are greater in gonadal males than gonadal females, is also influenced by the complement of sex chromosomes: the amount of vasopressin neural fibers is increased by the existence of the Y chromosome independently of gonadal sex (De Vries et al., 2002; Gatewood et al., 2006).

Regarding the LSi, the decrease in the number of LSi neurons in male rats by neonatal castration is prevented following injection with androstendione every other day during PD1-19 (Segovia et al., 2009). This finding suggests that androgens have an effect during the neonatal to prepubertal periods and are necessary for the formation of the male-typical structures of the LSi. The sex difference in LSi volume of rats is found on PD31, but not on PD16 (Tsukahara et al., 2004), indicating that this sex difference emerges during the prepubertal period between PD16 and PD31. Additionally, control of cell number by apoptotic cell death contributes to creating sex differences in cell number in several SDNs (Forger, 2009; Tsukahara, 2009). We found that the number of apoptotic cells in the male LSi is larger than that of the female LSi on PD16 (Tsukahara et al., 2004). The sex difference in the loss of cells by apoptosis during the late postnatal period is a contributing factor for producing the sex difference in neuron number of the LSi in adulthood.

#### Sex difference in the lordosis-inhibiting system in the LS

Development of the lordosis-inhibiting neural system is critical for defeminization of sexual behavioral patterns in male rats. In adult female rats, the actions of estradiol in the brain are a prerequisite for increasing sexual receptivity followed by lordosis display. However, estradiol, which induces lordosis in adult female rats, is ineffective in adult male rats, partially because the LS inhibits lordosis independently of estradiol (Satou and Yamanouchi, 1999). The LS of female rats also functions to inhibit lordosis, but this may be exhibited only in the anestrous phase, when the level of estradiol in blood is low. Direct implantation of E_2_ into the LS enhances the lordosis response in ovariectomized female rats that are subcutaneously treated with a low dose of EB in combination with progesterone (Satou and Yamanouchi, 1999). However, the treatment with EB and progesterone without E_2_-implantation is not sufficient for inducing the maximal level of sexual receptivity in ovariectomized rats (Satou and Yamanouchi, 1999). In contrast, direct implantation of E_2_ into the LS does not stimulate lordotic activity in castrated male rats receiving the same hormonal treatment (Satou and Yamanouchi, 1999). The number of Fos-expressing cells in the LS of female rats is increased by vaginocervical stimulation, and this increase in EB- and progesterone-treated ovariectomized rats is significantly smaller than that in vehicle-treated ovariectomized rats (Pfaus et al., 1996). This may support the possibility that the neuronal activity of the female LS, which is related to the inhibition of lordosis, is lowered by estradiol. Thus, it appears that female rats can be relieved of the inhibitory influence over lordosis by the direct actions of estradiol in the LS, while the lordosis-inhibiting function of the male LS cannot be released by estradiol. Moreover, it is reported that implantation of dihydrotestosterone into the LS inhibits lordosis in female rats (Tobet and Baum, 1982).

The mechanisms underlying the difference in the response of the LS to estradiol between the sexes and the resulting sex difference in inhibitory regulation of lordosis are yet to be shown. Long-term treatment with E_2_ increases the number of synapses in the LS of adult female rats, whereas this treatment fails to increase the number of synapses in the LS of adult male rats (Miyakawa and Arai, 1987), indicating a possible sexually dimorphic synaptic response to estrogens in the LS. Neural projections from the LSi to MCG, which are involved in the inhibition of lordosis (Tsukahara and Yamanouchi, 2001), are sexually dimorphic. The number of LSi neurons that were labeled by FG, a retrograde neurotracer, injected into the MCG is greater in female rats than male rats (Tsukahara and Yamanouchi, 2002; Tsukahara et al., 2003) (Figures 4A,B). The sex difference in the neural connectivity between the LSi and MCG is presumably related to the sex difference in inhibitory regulation of lordosis.

#### Formation of sexually dimorphic LS-MCG connections

Estradiol, which is locally synthesized in the brain from testosterone by aromatase in the perinatal period, affects the brain by masculinizing and defeminizing sexual behavioral patterns in rodents (McEwen et al., 1977; MacLusky et al., 1979; MacLusky and Naftolin, 1981). Injection of an anti-androgen drug or an aromatase inhibitor into pregnant rats enhances lordotic activity of offspring in both sexes in adulthood (Clemens and Gladue, 1978; Gladue and Clemens, 1978, 1982). Male rats castrated on the day of birth show lordosis behavior when they are treated with ovarian sex steroids in adulthood (Feder and Whalen, 1965). In contrast, treatment with testosterone propionate, an aromatizable androgen, on PD1 decreases lordotic activity in rats of both sexes (Gladue and Clemens, 1982). This effect of testosterone propionate may be mimicked by estradiol, because lordotic activity of female rats and neonatally castrated male rats is reduced by EB injection on PD5 (Whalen and Nadler, 1963; Feder and Whalen, 1965; Brown-Grant, 1975). Thus, defeminization of the neural systems regulating lordosis proceeds under the influence of aromatized testosterone originating from the testes during the perinatal period in rats.

The perinatal period, when aromatizable testicular testosterone is able to act as an agent for masculinization and defeminization of the brain, is not the sole stage but the initial stage of sex steroid-dependent sexual differentiation of the brain in rodents (Schulz et al., 2009a; Juraska et al., 2013). In the classic view of brain sexual differentiation, estradiol has been long considered to affect the brain during the perinatal period to masculinize and defeminize the brain. However, in addition to this action, estradiol was recently shown to play an active role in feminizing the brain during the prepubertal to adolescent period. Female aromatase knockout mice, which are deficient in the production of estradiol from testosterone, showed low levels of lordotic activity even after being treated with E_2_ and progesterone at adulthood (Bakker et al., 2002). The decreased lordotic activity in female aromatase knockout mice is recovered by injection of EB between PD16 and PD26, whereas EB injection between PD6 and PD16 has no effect (Brock et al., 2011). On the other hand, treatment with EB during PD6 to PD16 increases female-directed mounting behavior in testosterone-treated ovariectomized mice in adulthood, whereas there is no significant effect of EB treatment during PD16 to PD26 (Brock et al., 2011). In postnatal mice, the synthesis of estradiol in the ovary starts from PD7 (Mannan and O'Shaughnessy, 1991). Thus, the neural substrate involved in the regulation of lordosis is defeminized in males by estradiol originating from testicular testosterone during the perinatal period and conversely feminized in females by estradiol that is synthesized from the ovary during the late postnatal and prepubertal period. In males, testicular testosterone during the prepubertal period seems also to participate in the organization of sexual behavior. Indeed, prepubertal testosterone masculinizes and defeminizes sexual behavioral patterns in male hamsters (Schulz et al., 2004, 2009b).

The number of LSi neurons that were labeled by FG injected into the MCG is greater in ovariectomized female rats than castrated male rats with or without E_2_ treatment at adulthood (Tsukahara and Yamanouchi, 2002). This indicates that sexually dimorphic neural connectivity between the LSi and MCG is not influenced by estradiol in adulthood. In contrast, estradiol in the postnatal period is a key molecule for the sexually dimorphic formation of LSi-MCG neural connections. The number of LSi neurons projecting to the MCG in female rats is dose-dependently decreased by treatment with EB on PD5 (Tsukahara et al., 2003) (Figures 4A,B). Moreover, this hormonal treatment decreases lordotic activity of adult female rats in a dose-dependent fashion (Figure 4C). Performance of lordosis in adult female rats can be decreased by neonatal treatment with an ERα agonist, but not an ERβ agonist (Patchev et al., 2004; Kanaya and Yamanouchi, 2012). The LSi of postnatal rats expresses ERα but not ERβ (Perez et al., 2003). Thus, defeminization of sexual behavior partly results from the development of the male LS, which contains less neuron projecting axons to the MCG than the female LS and inhibits lordosis independently of estradiol, under the influence of aromatized testosterone binding with ERα during the postnatal period. In contrast to estradiol originating from testicular testosterone during the postnatal period, estradiol that is secreted from the ovaries before puberty acts to feminize the neural substrate regulating lordosis behavior (Brock et al., 2011). Ovarian estradiol during the prepubertal period may contribute to the development of the female LS, which contains many more neuron projecting axons to the MCG than the male LS, resulting in the release from the inhibitory influence of the LS on lordosis in female rats by the direct action of estradiol at adulthood. The effect of prepubertal estradiol on the formation of sexually dimorphic LSi-MCG neural projection remains to be investigated.

In addition to sex steroids, genetic differences between males having a single X chromosome and a Y chromosome and females having two X chromosomes, which could cause sex-specific gene expression in the brain independently of sex steroids, is a sex-biasing factor in behavioral expression (Cox et al., 2014). Steroidogenic factor 1 (SF-1) is a transcriptional factor required for gonadal development, and therefore SF-1 knockout mice of both sexes are not exposed to endogenous gonadal steroids and have female external phenotypes regardless of genetic sex (Ingraham et al., 1994; Grgurevic et al., 2012). According to one report, lordotic activity was drastically decreased in EB- and progesterone-treated SF-1 knockout mice in comparison to wild-type ovariectomized female mice that bore the same hormonal treatment (Grgurevic et al., 2012). However, there was still a sex difference in lordotic activity in SF-1 knockout mice, and the LQ of SF-1 knockout female mice was significantly higher than that of SF-1 knockout male mice (Grgurevic et al., 2012). This finding suggests that sex chromosome effects partly contribute to the sexual differentiation of sexual behavioral patterns. Genes of the X chromosomes may promote behavioral feminization, or genes of the Y chromosome may induce behavioral defeminization of mice.

The mechanisms responsible for postnatal estradiol-dependent, sexually dimorphic formation of the LSi-MCG neural connections remain to be investigated. There are several potential mechanistic explanations. On PD16, male rats have a significantly greater number of apoptotic cells in the LSi than female rats (Tsukahara et al., 2004). The sex difference in postnatal apoptosis may be partially related to formation of the sexually dimorphic LSi-MCG neural connection. However, not all neurons killed by apoptosis during the postnatal period may be fated to project axons to the MCG, even if they survive and elongate axons. Moreover, sex differences in apoptosis arise on PD16, after PD5 when EB exhibits significant effects on the sexually dimorphic formation of the LSi-MCG neural connection. Epigenetic changes in the developing brain are caused by transitory exposure to estradiol during the perinatal period. The epigenetic changes caused by estradiol exhibit long-lasting effects, inducing permanent sex differences in the morphology and function of the brain (Nugent et al., 2011; Matsuda et al., 2012). Although the time lag between EB actions and sex differences arising in apoptosis may be explained by epigenetic regulation by estradiol, further studies are needed to clarify the involvement of postnatal apoptosis on the estradiol-dependent sexually dimorphic formation of LSi-MCG neural projections. It is also possible that sex differences in LSi-MCG neural projections are due to a sex difference in terminal arborization of LSi neurons at the MCG. The female LSi would contain many more FG-labeled neurons than the male LSi if terminal arborization of LSi neurons projecting to the MCG showed more complexity in female rats. However, there is no evidence for sex differences in the terminal arborization in the MCG. One report shows that daily subcutaneous injections of EB for 20 days increases the number of nerve terminals and synapses in the MCG of ovariectomized rats (Chung et al., 1988). This report suggests that estradiol increases synaptic plasticity in the MCG of female rats. However, sexually dimorphic neural connectivity between the LSi and MCG is not influenced by estradiol in adulthood (Tsukahara and Yamanouchi, 2002).

Several lines of evidence indicate that estradiol modulates axon outgrowth. Axon outgrowth of cultured neurons originating from the fetal hypothalamus is promoted by E_2_ (Cambiasso et al., 2000; Carrer et al., 2005). The neural projection from the BNSTp to the AVPV exhibits a sex difference. It is more prominent in male rats and this sex difference is dependent on the effects of testosterone in the AVPV in the postnatal period (Ibanez et al., 2001). This suggests that testosterone, or its metabolite estradiol, induces production of a chemotrophic factor at the target of neural projections. This chemotrophic factor directs the innervation by projection neurons to produce the sexually dimorphic neural projections. It is also known that estradiol has opposite effects on axon outgrowth. The density of mesencephalic serotonergic fibers in the medial preoptic nucleus of male rats is lower than that of female rats, and perinatal treatment with testosterone propionate masculinizes the serotonergic fibers in female rats (Simerly et al., 1985). Neurite growth of cultured serotoninergic neurons, which originate from the mesencephalon of rat embryos and express ERα and ERβ, is inhibited by EB (Lu et al., 2004). Both the LSi and MCG during the postnatal period are presumably the action sites of estradiol, because ERα is expressed in both regions in postnatal rats (Perez et al., 2003). Neurons of the LS elongate axons to the MCG during the period between PD5 and PD15, and most of the axons complete termination at the MCG on PD15 (Kouki and Yamanouchi, 2007). Aromatized testosterone originating from the testes in the perinatal period may affect the LSi and/or MCG to suppress axon outgrowth from the LSi to MCG, resulting in the formation of sexually dimorphic neural connections between the LSi and MCG.

## Summary and future directions

The LS plays an inhibitory role in the regulation of lordosis in rats of both sexes. For male rats, the LS is important for suppressing heterotypical sexual behavior. For female rats, the LS is important for suppressing sexual behavior in the anestrous phase. The LS functionally and anatomically links to the MCG, but not to the VMN, in the inhibitory regulation of lordosis. Lordosis-inhibiting neurons are located in the LSi, and they project axons to the MCG. The neural connection between the LSi and MCG is sexually dimorphic. There are greater numbers of LSi neurons projecting to the MCG in female rats than in male rats. The inhibitory regulation of lordosis by the LS differs between sexes with respect to responsiveness to estradiol: female rats can be relieved of the inhibitory influence on lordosis by the direct actions of estradiol in the LS, while inhibition of lordosis by the LS cannot be released by estradiol in male rats. Sexually dimorphic neural connections between the LSi and MCG form the morphological basis of the sex difference in the inhibitory regulation of lordosis by the LS. Further studies are needed to examine estrogen signaling in the LSi, and how this modulates activity of lordosis-inhibiting neurons in female rats. Additionally, how LSi neurons project to the MCG and inhibit lordosis independently of estradiol in male rats needs to be investigated. Defeminization of sexual behavioral patterns by estradiol during the postnatal period may partly result from the sexual differentiation of the LS. Estradiol in the postnatal period serves as a key molecule to form male-like LSi-MCG neural pathways. However, it remains to be determined which mechanisms form these sexually dimorphic LSi-MCG neural projections and how prepubertal estradiol contributes to the formation of female-like LSi-MCG neural connection. Accumulating evidence has provided further insights on the control of sexual behaviors in rodent models. However, the mechanisms responsible for the inhibitory regulation of lordosis are less well understood. Therefore, further studies are needed to better our understanding of female reproduction and sexual differentiation of the brain.

### Conflict of interest statement

The authors declare that the research was conducted in the absence of any commercial or financial relationships that could be construed as a potential conflict of interest.
